# Hypertension Diagnosis with Backpropagation Neural Networks for Sustainability in Public Health

**DOI:** 10.3390/s22145272

**Published:** 2022-07-14

**Authors:** Jorge Antonio Orozco Torres, Alejandro Medina Santiago, José Manuel Villegas Izaguirre, Monica Amador García, Alberto Delgado Hernández

**Affiliations:** 1 TecNM, Campus Tuxtla Gutiérrez, Carretera Panamericana Kilometro 1080, Tuxtla Gutiérrez 29050, Chiapas, Mexico; 2 National Science and Technology Council (Conacyt), Department of Computer Science, National Institute for Astrophysics, Optics and Electronics, San Andrés Cholula 72840, Puebla, Mexico; 3 Facultad de Ciencias de la Ingeniería y Tecnología, Universidad Autónoma de Baja California, Boulevard Universitario #1000, Unidad Valle de las Palmas, Tijuana 21500, Baja California, Mexico; villegas_josemanuel@uabc.edu.mx (J.M.V.I.); delgado.alberto@uabc.edu.mx (A.D.H.); 4 Center for Technological Research, Development and Innovation, University of Science and Technology Descartes, Tuxtla Gutiérrez 29065, Chiapas, Mexico; 5 TecNM, Campus RioVerde, Carretera Rioverde-San Ciro Kilometro. 4.5, Rioverde 79610, San Luis Potosi, Mexico; mony_951@hotmail.com

**Keywords:** backpropagation neuronal network, artery hypertension, health diagnosis, public health, sustainability

## Abstract

This paper presents the development of a multilayer feed-forward neural network for the diagnosis of hypertension, based on a population-based study. For the development of this architecture, several physiological factors have been considered, which are vital to determining the risk of being hypertensive; a diagnostic system can offer a solution which is not easy to determine by conventional means. The results obtained demonstrate the sustainability of health conditions affecting humanity today as a consequence of the social environment in which we live, e.g., economics, stress, smoking, alcoholism, drug addiction, obesity, diabetes, physical inactivity, etc., which leads to hypertension. The results of the neural network-based diagnostic system show an effectiveness of 90%, thus generating a high expectation in diagnosing the risk of hypertension from the analyzed physiological data.

## 1. Introduction

In the 20th century, cardiovascular diseases underwent a very important change, from being an infrequent cause of death and disability at the beginning of the century, to being considered a major factor in death and permanent damage throughout the world at the end of the century. According to data from the World Health Organization (WHO), in 2001, the main cause of death in the adult population was cardiovascular disease in five of the six regions suggested by the WHO; that is, 30% of these deaths were related to this health factor, leading to a total of 17 million deaths. This increase in the mortality rate in a relatively short period of time is mainly due to changes in diet, sedentary lifestyles and the increase in life expectancy, which is part of the development of societies in an industrialized environment. The high prevalence of hypertension in various populations has significantly generated a wave of diseases, and therefore, it is estimated that five million deaths present cerebral vascular events, which is an indicator of the presence of hypertension [1,2,3,4].

According to the WHO and the World Bank, in their recent reports, hypertension is considered a barrier to a healthy population, mainly because of its high prevalence in the world, particularly in low-income and middle-income countries [5].

The main obstacles to blood pressure control are based on poor management strategies in primary health care. In addition, the socioeconomic situation of the population varies from country to country and, in some cases, from region to region within the country. This is coupled with the epidemiology of hypertension and related diseases, resources, and health care priorities [6].

It is a serious condition that significantly increases the risk of heart disease, encephalopathy, kidney disease and other diseases. An estimated 1130 million people worldwide have hypertension, and most of them live in low- and middle-income countries (about two-thirds). In 2015, 1 in 4 men and 1 in 5 women had high blood pressure. Only 1 in 5 people with high blood pressure have it under control. Hypertension is one of the leading causes of premature death in the world. One of the global targets for non-communicable diseases is to reduce the prevalence of hypertension to 25% by 2015 (from baseline values reported in 2010 [7]).

In Latin America and the Caribbean, between 20% and 35% of the adult population is considered to have hypertension. In a study conducted in four South American countries (Argentina, Chile, Colombia and Brazil) [6], 57.1% of the estimated adult population with hypertension knows that they are hypertensive, and, in recent years, this factor has increased and some people are unaware of this condition. This shows the low level of control of the population; only 18.8% of hypertensive adults in these four countries have their blood pressure under control. It is advisable to prevent and treat in a timely manner to maintain blood pressure levels below 140/90 mmHg, since hypertension, as such, is not curable. Adequate control can prevent premature deaths, considering the population with uncontrolled hypertension, suboptimal blood pressure and the untreated, in a period of 10 years, this could prevent 10 million deaths in the world due to cardiovascular factors. PAHO/WHO works to improve hypertension control programs, promoting policies for its prevention and projects that contribute to the training and updating of health personnel, especially at the primary care level [8]. Recent data recorded in China and Singapore showed that between 12.8% and 31.2% of patients with COVID-19 had pre-existing hypertension, representing an important comorbidity in SARS-COV-2-infected patients. These patients appeared to develop the disease more frequently and were more susceptible to death [9,10].

In Mexico, full efficiency has not been achieved at the first level of medical care, whose maximum sign of effectiveness should be to prevent the flood of patients who could have been controlled or limited in their progression of complications to hospital levels of care.

The main motivation that led to conducting this research is the need to diagnose hypertension through the application of ANN. This method is an innovative alternative and considered a sustainable development project that seeks the satisfaction of diagnosing without compromising future needs, whose result and implementation will generate a benefit, taking as initial information the health levels of the student community of the School of Science and Technology UABC (ECITEC).

Currently, several works have been reported related to the use of neural networks, including Yolanda García Montero [11], as well as Zainab Assaghir et al. [9], and the short article presented by Arpneek Kaur and Abhishek Bhardwaj [12]. In each, different methodologies of analysis are presented that differ from what is proposed in this research.

The article is structured as follows. Section 2 presents the data collection, divided into subsections such as problem statement, justification, hypothesis and theoretical framework; Section 3 defines the design methodology, which entails a brief description of neural networks, their structure and neural architecture, all as part of the steps for the research; Section 4 addresses the numerical results of the diagnostic system, the pseudo-code of the neural network, network training, network training state characterizing the system output and regression training; Section 5 presents the neural architecture classification tests, which aim to provide data related to information processing and the results obtained. Section 6 presents the discussion of the results obtained, and finally, Section 7 presents the conclusions of the work as well as future work.

## 2. Data Collection

### 2.1. Problem Statement

One in four Mexicans suffers from hypertension; in men, the prevalence is 24.9%, and in women, it’s 26.1%. The estimated prevalence is 30% according to the criteria of 140/90 mm Hg, which is equivalent to about 30 million corresponding to the diagnosis of hypertension. However, with the new criteria of the American Heart Association, the population with hypertension will be at least double [13,14].

According to the 2016 National Survey of Health and Nutrition Midway 2016 (ENSANUT 2016), conducted by the National Institute of Public Health (INSP) and the Ministry of Health of the federal government, one in four adults in Mexico suffers from high blood pressure; that is, 25.5 percent of the population, of which approximately 40 percent are unaware that they have this disease, and this impacts their health condition. Only about 60 percent know their diagnosis, and only half of those are controlled [15].

According to the National Health and Nutrition Survey 2020 on COVID-19 Organ under the National Institute of Public Health of the Federal Government of Mexico, the prevalence of hypertension by previous medical diagnosis in the adult population aged 20 years and older was determined in 2012, 2016, 2018 and 2020. The prevalence of hypertension was 13.4% in 2020, which is lower than previous surveys (16.6% in 2012, 15.3% in 2012 and 18.4% in 2018), but was not statistically different compared to 2016. In women, the prevalence of hypertension by medical diagnosis increased from 18.5% to 20.9% from 2012 to 2016, but the prevalence decreased to 15.7%, increasing from 18.5% to 20.9% from 2012 to 2016, but the prevalence decreased to 15.7% in 2020. In men, a smaller increase in prevalence was observed from 2012 to 2016 (from 14.1 to 15.3%), and in 2020, the estimated prevalence was 10.9% [16].

### 2.2. Justification

Neural networks are considered within Artificial Intelligence (AI), whose application has revolutionized many sectors, medicine being one of them. Currently, there have been several research papers related to measuring or diagnosing the risk of hypertension using neural networks. Therefore, and in function to the bibliographic search of these investigations, the necessity is born to approach as a new alternative the use of diverse physiological aspects, whose information and with the implementation of a neural network is able to diagnose the risk of suffering hypertension.

### 2.3. Hypothesis

The use of neural networks has been very evolutionary in recent years, covering various areas of knowledge, including medicine, whose use ranges from the prognosis of the evolution of different pathology’s or therapeutic interventions, classifying or recognizing anatomopathological samples, imaging tests or establishing diagnostic probabilities from symptoms or complementary tests [1].

Therefore, we dare to say that the model for the diagnosis of hypertension can be a tool for further application in medicine, whose study, design and experimentation are essential for the expected results.

### 2.4. Theoretical Framework

**Figure 1:**
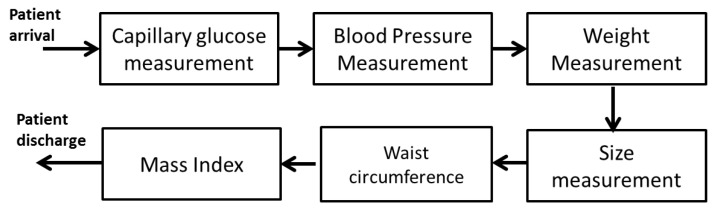
Sequence of the procedure for taking the medical variables to be used.

The population analyzed includes all students in ECITEC currently enrolled in their study programs, which were sampled (medical variables), represented through a block diagram (Figure 1). The procedure for taking the medical variables is indicated, and Table 1 indicates the limits considered in each measurement process from the medical point of view.

Samples were taken from students, and were recorded in an Excel sheet containing the student’s name, sex, age, glucose level, blood pressure, weight, height, waist (cm), and body mass index. Subsequently, the information was analyzed and classified for processing.

## 3. Design Methodology

### 3.1. Neural Networks

Artificial neural networks are non-linear approximations to the way the brain works; therefore, they should not be directly compared to the brain, nor should the principles underlying the functioning of artificial neural networks and the brain be confused, nor should it be thought that neural networks are based solely on biological networks, since they only emulate the functioning of the human brain in a very simple way. Furthermore, it should be considered that biological networks are generators of neurobiological processes in which highly complex relationships are established, which cannot be achieved with monolayer or multilayer networks. ANNs can be studied as universal approximators from a mathematical point of view. The following is an overview of the biological foundations of natural neural networks, without going deeper into this exciting field, as learning about such a complex subject requires many other volumes to study. Only a basic idea is developed here that serves as a primary approach to the study of artificial neural networks.

**Figure 2:**
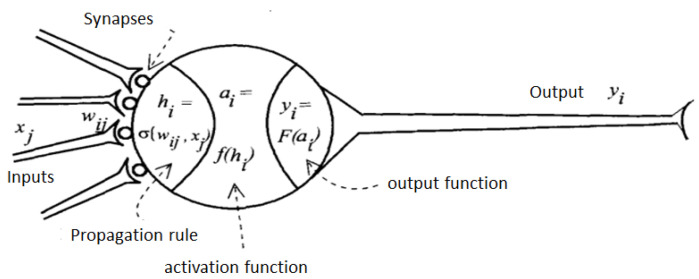
Generic model of artificial neuron.

Neural networks are a simulation of the biological diversity properties of neural systems by means of mathematical models recreated through artificial mechanisms (an integrated circuit or a computer) [5,16,17]. The aim is to have machines give responses similar to those that the human brain is capable of giving. An elementary processor or neuron is a simple computational device that, from an input vector coming from the outside or from other neurons, provides a single response or output. The element that constitutes a neuron is shown in (Figure 2). A neural network is characterized by the following elements:

- A set of processing units or neurons.
- An activation state for each unit, equivalent to the output of the unit.
- Connections between the units, usually defined by a weight that determines the effect of an input signal on the unit.
- A propagation rule, which determines the effective input of a unit from external inputs.
- A trigger function that updates the new trigger level based on the effective input and the previous trigger.
- An external input corresponding to a term determined as bias for each unit.
- A method for gathering the information, corresponding to the learning rule.
- An environment in which the system will operate, with input signals and even error signals.

The mathematical model of the above-mentioned biological neuron proposed by McCulloch and Pitts [18], usually called an M-P neuron. In this model, the *i*th processing element computes a weighted sum of its inputs and outputs $y_{i}$ = 1 (firing) or 0 (not firing) according to whether this weighted input sum is above or below a certain threshold $\theta_{i}$:

(1) $$\begin{array}{c} y_{i}\left(t+1\right)=a\left(\sum_{j=1}^{m}w_{ij}x_{j}\left(t\right)-\theta_{i}\right) \end{array}$$

where the activation function *a*(*f*) is a unit step function:

(2) $$\begin{array}{c} a\left(f\right)=\left\{\begin{array}{c} 1\;if\;f\geq 0\\ 0\;otherwise \end{array}\right. \end{array}$$

### 3.2. Structure

A multilayer feed-forward neural network is one of the first efforts to represent supervised learning, where elementary activation functions are used in binary form. In most cases, the MLP network consists of an input layer [19], one or more hidden layers and an output layer. When using feed-forward neural networks, it is required that there is an adjustment of the weights associated with the connections, so that there is a learning by the network. To perform this weight adjustment there are many techniques, among which is the Backpropagation Algorithm or Backpropagation error [20].

**Figure 3:**
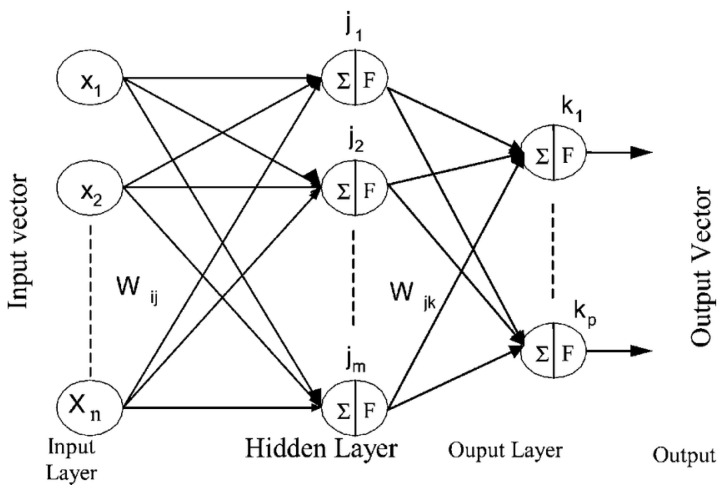
Multilayer perceptron architecture with backpropagation algorithm.

The Backpropagation algorithm is a supervised training used for multilayer networks, where the value of the weights is adjusted according to the error generated. This technique is widely used as it allows an optimization method to be found by defining the error gradient and minimizing it with respect to the neural network parameters. The structure of the backpropagation neural network algorithm [21] is shown in (Figure 3).

Algorithm Back-propagation Learning Rule [22,23].

The first step is to propagate the input forward through the network:

(3) $$a^{0}=p,$$

(4) $$a^{m+1}=f^{m+1}\left(w^{m+1}a^{m}+b^{m+1}\right)form=0,2,...,M-1$$

(5) $$a=a^{M}$$

The next step is to propagate the sensitivities backward through the network:

(6) $$s^{M}=-2F^{M}\left(n^{M}\right)\left(t-a\right),$$

(7) $$s^{m}=F^{m}\left(n^{m}\right){\left(W^{m+1}\right)}^{T}s^{m+1},form=M-1,...,2,1,$$

Finally, the weights and biases are updated using the approximate steepest descent rules:

(8) $$W^{m}\left(k+1\right)=W^{m}\left(k\right)-\alpha s^{m}{\left(a^{m-1}\right)}^{T},$$

(9) $$b^{m}\left(k+1\right)=b^{m}\left(k\right)-\alpha s^{m}.$$

### 3.3. Proposed Neural Architecture

**Figure 4:**
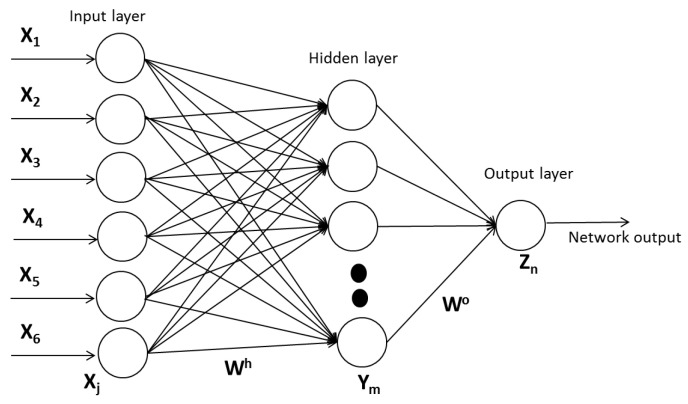
Multilayer feed-forward neural network.

The proposed feed-forward multilayer neural network with backpropagation learning algorithms is shown in (Figure 4) [24].

The Backpropagation learning algorithm its characterized by Equation (10):

(10) $$\begin{array}{c} a^{3}=f^{3}(w^{3}f^{2}\left(w^{2}f^{1}\left(w^{1}\left(p+b^{1}\right)\right)+b^{2}\right)+b^{3} \end{array}$$

The characteristic equation of the development of the system for the diagnosis of hypertension is:

Diagnostic System =

W1=((Edad∗P11)+(DxTx∗P12)+(FC∗P13)+(FR∗P14)+(Tem∗P15)+

(Sistolica∗P16)+(Fuma∗P18)+(IMC∗P19)

$A1=tansig(W1+b_{1}^{1})$

W2=(((A1∗P21)+(A1∗P22)+(A1∗P23)+(A1∗P24)+(A1∗P25)+(A1∗

P26)+(A1∗P27)+(A1∗P28)+(P1∗P29))+((A1∗P210)+(A1∗P211)+…+

(A1∗P218))+((A1∗P219)+(A1∗220)+…+(A1∗P227))+((A1∗P228)+(A1∗

P229)+…+((A1∗P236)))

$A2=tansig(W2+b_{1}^{2})$

W3=(((A2∗P31)+(A2∗P32)+(A2∗P33)+(A2∗P34))+((A2∗P35)+(A2∗

P36)+(A2∗P37)+(A2∗P38)))

$A3=logsig(W3+b_{1}^{3})$

## 4. Results

The data in Table 2, are a fragment of the original, composed of 303 records as part of the sample population of a total of 2500 peoples, which must be read punctually to arrive at the results through the proposed neural network.

Table 3 shows expected results against the simulated data through the network. There was a great similarity of the data between those expected to be obtained and those obtained by the proposed neural network, which were grouped by class in relation to them, which allows affirming that there is certainty in the results themselves.

The following Matlab expressions (a to d) show the numerical results of the diagnostic system using the data sheet.

- A1 = [21 174 84 15 24.7 94]; a1 = sim(net,A1) a1 = 0.2819 (a)
- A2 = [20 118 75 21 34.6 122]; a2 = sim(net,A2) a2 = 0.3721 (b)
- A3 = [18 103 77 18 19.4 130]; a3 = sim(net,A3) a3 = 0.7439 (c)
- A4 = [22 111 70 23 30.5 140]; a4 = sim(net,A4) a4 = 0.8682 (d)

**Figure 5:**
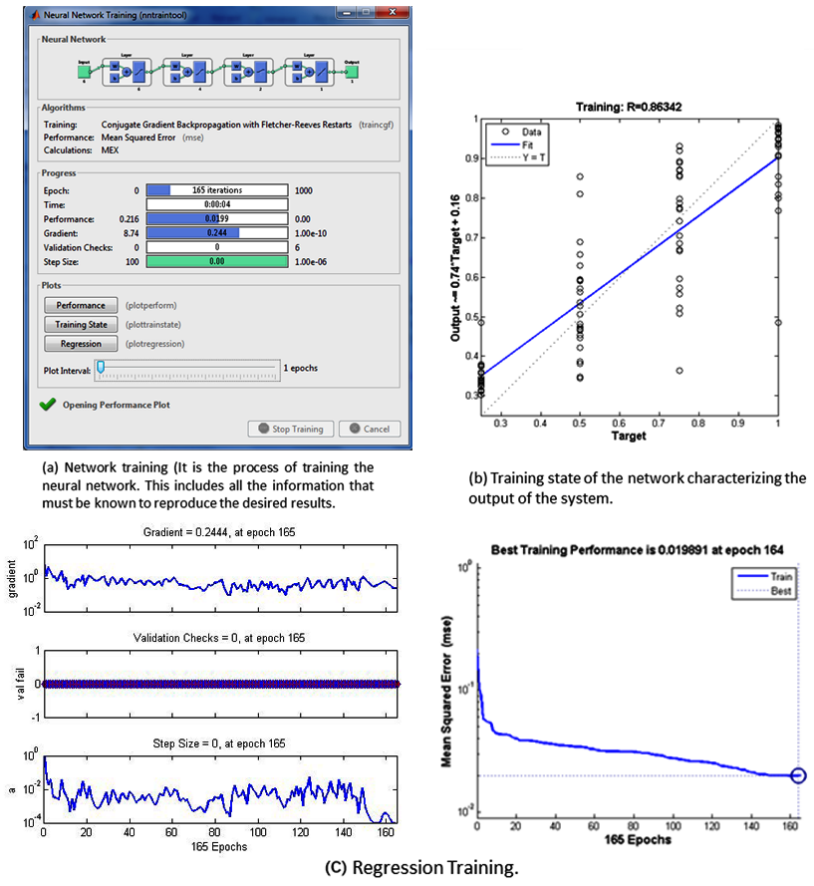
(**a**) shows the training of the neural network, (**b**) shows the state of the network training, and (**c**) shows the behavior of the network in its training with the regression method.

In the Figure 5a shows the training of the neural network, Figure 5b shows the state of the network training, and Figure 5c shows the behavior of the network in its training with the regression method.

**Figure 6:**
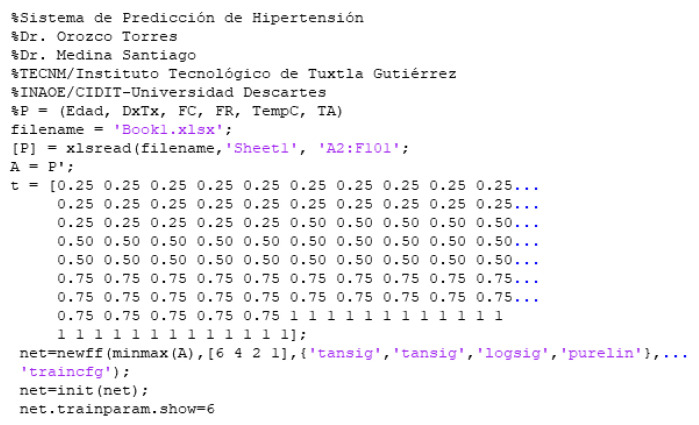
Diagnostic system pseudocode.

Figure 6 shows the pseudo-code for the neural network information processing with backpropagation learning. Where we can observe the lines of code that make up the simulation and processing of the data, to obtain results.

## 5. Neural Architecture Classification Tests

**Figure 7:**
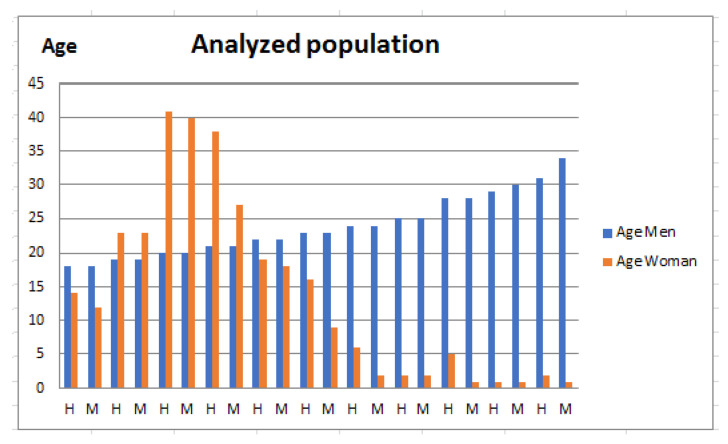
Age of the population analyzed.

This section presents complementary information that allows us to understand the proposed method. Figure 7 presents the statistics of the population attended, which shows a homogeneity of age in the population. As can be seen, the segment of the population analyzed is relatively young, even so, it is important to mention that hypertension has been detected in both young and adult populations.

Our population comprises all students of the different careers offered at ECITEC (School of Science and Technology UABC) located in Baja California, Norte Mexico, which, in 2018, was more than 2500 people. Participation in this project was voluntary for the morning and afternoon shifts, which are the shifts of attention to the population.

Table 4 shows the information sheet generated from the students’ participation. This information is the basis for feeding the proposed neural network, based on the information, a classification of the level of hypertension was carried out and marked with colors according to the parameters presented in Table 5. Table 4 is only a fragment of all the information processed.

Table 6 shows the information generated from the analysis of the information through the neural network proposed for this work. Due to its large size, only a fragment of it is presented.

### Results Obtained

This section shows the effects on the neural architecture proposed for the classification of the data obtained. Table 7 shows the different classes for systolic pressure considered in the ANN data analysis.

**Figure 8:**
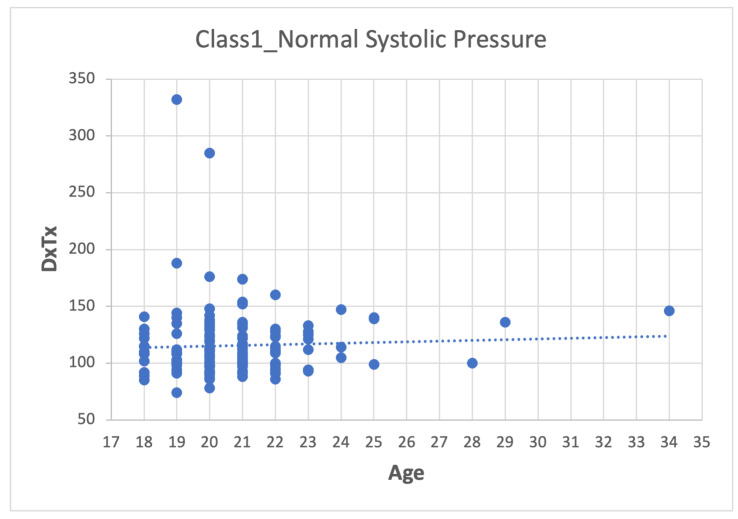
Age-DxTx graph.

Depending on the dataset, data pre-processing can represent between 10–60% of the time and effort for the data mining process [26,27,28]. The method used was:

1. First step: division of the database according to the categories presented in Table 7, which leads to four datasets (Class_1, Class_2, Class_3 and Class_4).
2. Second step: Analyze the data for each of the variables in each class using scatter plots. For category Class1_Systolic Pressure Norm, we obtain:
  Figure 8, showing two outliers that were analyzed and considered as normal due to the nature of the variable. On the other hand, we observe that within the variable Age, we also had values far to the right, but these corresponded to students whose age was older than the majority.

**Figure 9:**
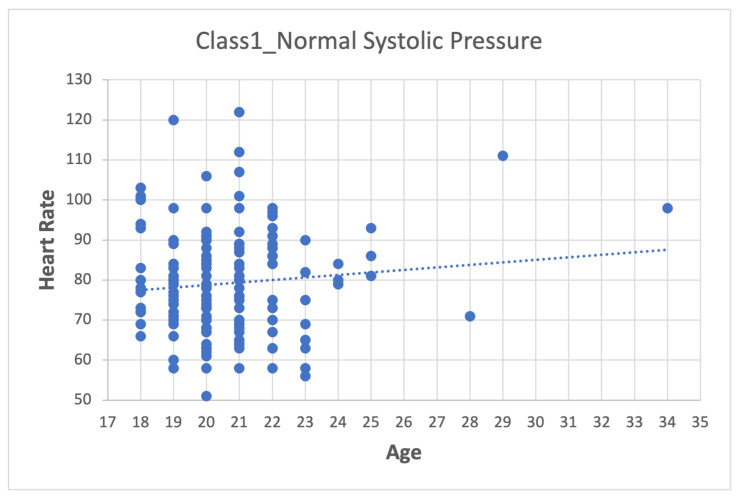
Age–Heart Rate graph.

Figure 9 shows heart rate (HR) values within the normal range, with no values atypical.

**Figure 10:**
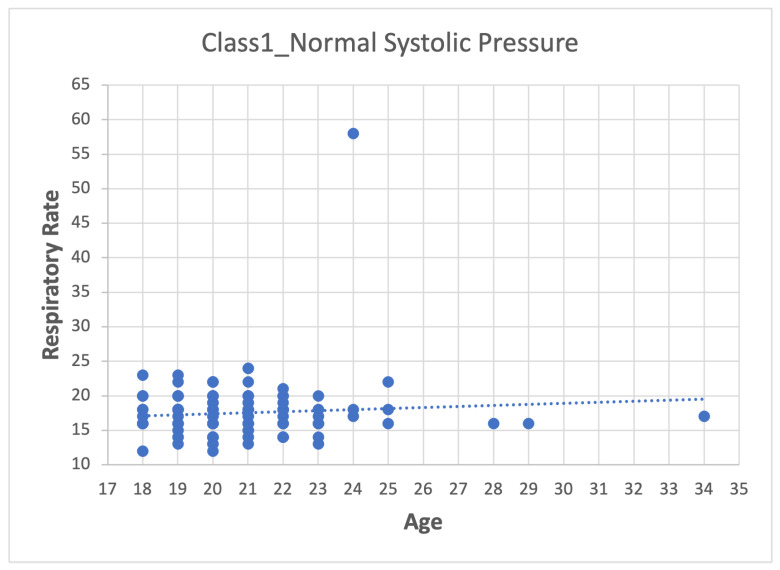
Age–Respiratory Rate graph.

Figure 10 shows the respiratory frequency (RF) with a single values atypical.

**Figure 11:**
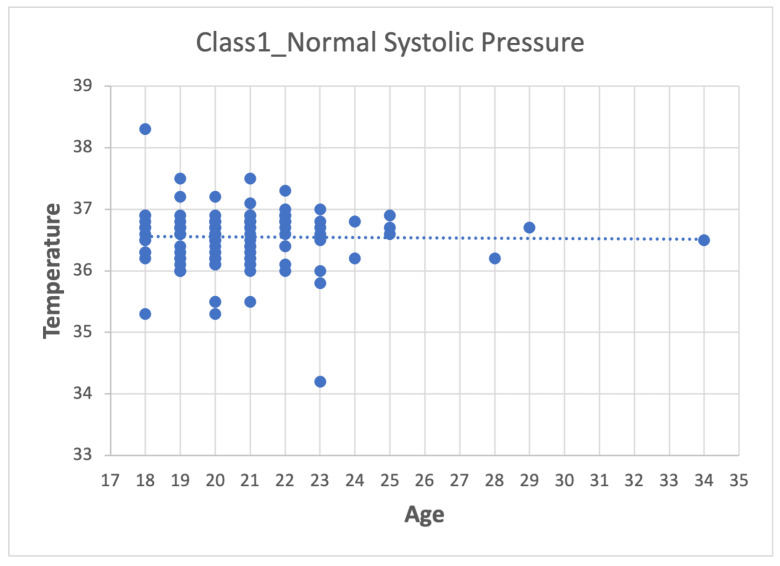
Age–Temperature graph.

Figure 11 shows temperature values (°C) without values atypical according to the nature of the medical variable.

**Figure 12:**
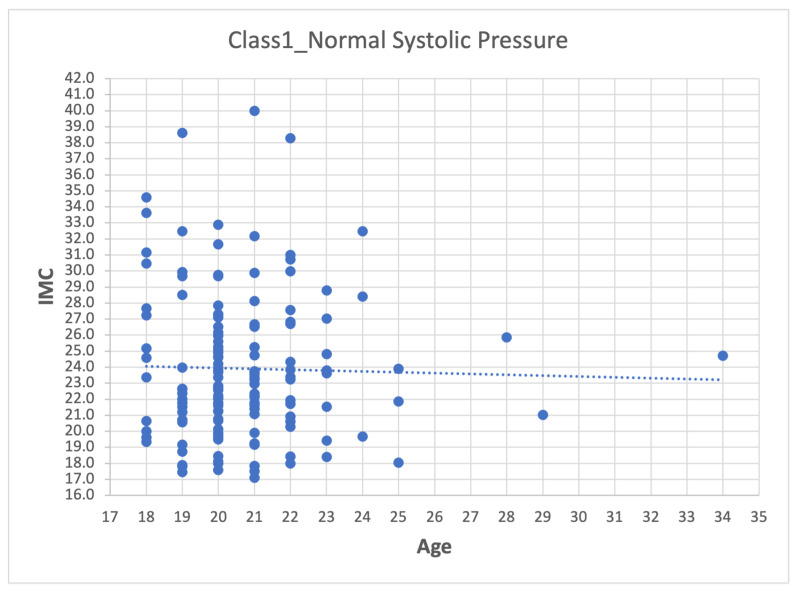
Edad–IMC.

Figure 12 Represents BMI (Body Mass Index) data, which is a variable that is closely related to the weight of a person and is considered important for the processing of information with the neural network.

3. Third step, correlation analysis of Class1_Systolic Normal Pressure variables is shown in Table 8 below.

Table 8 highlights the highest correlation values for the Class1_Systolic Normal Pressure variables and we recall that when the correlation is positive, this indicates that its linear regression projection will tend to increase together with the counter variable. Conversely, when the correlation is negative, this indicates that the linear regression projection will tend to decrease along with the counter variable. It is important to note that during the application of the correlation in the different classes we always obtained the same values as in Table 7. It should be noted that we have followed the same steps, for each variable in each of the different categories set out in Table 7.

Table 9 and Table 10 represent obtained classification values of the neural architecture with the learning algorithms using the data obtained from the sample. With final results obtained from the neural network architecture, it is shown that the classification is acceptable for class_2 and class_3; with respect to class_1 and class_4 the classification percentages are very optimal.

## 6. Discussion

The discrepancy between the results of this study with respect to the work done by Assaghir, Zainab and Janbain, Ali and Makki, Sara and Kurdi, Mazen and Karam, Rita [29] (using Neural Network to predict Hypertension in 2017), may be because this is the first where the population under study belongs to several universities in the country of Lebanon, and takes into account variables such as stress, gender among others. In our case, our study is limited to the student population of ECITEC. It should be added that our aim was to classify the cases of hypertension in its different levels, as indicated at the beginning of the article. In the short term, this type of diagnosis could be applied to assign a driver’s licence to a car user, but also to generate a database for car insurers to determine the physical conditions of the driver(s) to reduce risk for other drivers in the social and urban environment where the instrumented driver is located.

The methodology used for the development of the algorithm was carried out in the following stages: data acquisition, analysis of each variable, principal component analysis of the database and development of the Backpropagation Neural Network, using Microsoft Excel and Minitap software as tools for the exploration of the data before using them in the artificial neural network. A database of medical signals was obtained; the database allows the training of the artificial neural network for learning. The classification of the backpropagation neural network showed better results using the algorithm with the “feedforwardnet” instruction than using “newff”. The classification results for Class_1, Class_2 and Class_4 were satisfactory, but the results for Class_3 need to be improved.

There are several techniques for the diagnosis of hypertension among them SVM, Navie Bayes, which we analyzed and compared, adding the following comparatives Table 11 and Table 12.

## 7. Conclusions

The results of the neural network-based diagnostic system show an effectiveness of 90%, thus generating a high expectation in diagnosing the risk of hypertension from the analyzed physiological data.

An explanation of the results contained in this article is that the problem of hypertension goes beyond the school environment that leads to various environments, such as home and community, where physiological aspects are altered. Then hypertension ceases to be a personal and family matter, becoming a public health problem that requires the coordinated support of all social sectors. The study and analysis of hypertension is now well regarded by researchers developing safety algorithms to improve the comfort and safety of driving a car by a hypertensive person.

Arterial hypertension (AHT) [38] is the most prevalent chronic disease in developed countries and its etiology is unknown in 90–95% of patients. Its progression can lead to very serious complications for the patient. Essential hypertension is a multi-causal and heterogeneous disease, which is related to hereditary or genetic factors and to sodium-rich diet, stress, smoking, sedentary lifestyle and obesity. HTN is associated with atherosclerosis and its complications such as the heart, kidney, brain; such as hypertensive cardiomyopathy, myocardial ischaemia, heart failure, ventricular arrhythmia, sudden death, renal failure, aortic aneurysm, peripheral arterial disease, cerebral infarction due to thromboembolism and cerebral hemorrhage. All these complications are serious and disable conduction for a long period of time, and, in many cases, permanently. The development and progression of AHT varies widely from patient to patient, so driving advice should be tailored to the individual driver’s characteristics.

Drivers with AHT should avoid risk factors that promote it, such as a diet rich in salt, a sedentary lifestyle, obesity, smoking, hypercholesterolemia and stress. Following these tips will also help you to drive more comfortably. Patients with established visceral damage or severe, refractory or malignant hypertension, or with evidence of hypertensive encephalopathy, should not drive. Urgent hypertensive crisis and hypertensive emergency prevent driving until the patient is perfectly controlled without involvement of visceral lesions that impair their ability to drive. In general, driving is not advisable in symptomatic hypertensive patients and/or those refractory to medical treatment, as these situations make driving more difficult and increase the risks at the wheel. If a hypertensive patient notices symptoms while driving, they should stop as soon as possible and park the vehicle in an area where there is no risk of accident, then calm down and wait for the symptoms to subside. A hypertensive patient with symptoms cannot drive; if they do not improve, they should ask for help to be transferred to the nearest health centre. The favorable evolution of the different situations with the specific treatment for adequate BP control will allow the physician to assess the patient’s ability to drive.

## Figures and Tables

**Table 1:** Limits considered in each medically permissible measurement process.

Measurement Type	Type	Limits
Glucose	No diabetes	Before meals 70–110 mg/gL
		After meal < 140 mg/gL
	With diabetes	Before meals 80–130 mg/gL
		After meal < 180 mg/gL
Blood Pressure	Systolic (Highest value)	90 or lower hypotension
		91 to 119 normal
		between 120 and 129 high
		between 130 and 139 stage 1 hypertension
		140 or higher stage 2 hypertension
		greater than 180 hypertensive crisis
	Diastolic (lowest value)	60 or lower hypotension
		61 to 79 normal
		and less than 80 high
		or between 80 and 89 stage 1 hypertensions
		or 90 or greater stage 2 hypertension
		greater than 120 hypertensive crisis
Weight	Variations in observation may be due to sex, age of the individual and many other factors.	
Body Mass Index (Quetelet Index)	BMI = Weight (Kg)/Height (m)${}^{2}$	Numbers less than 18 indicate low weight.
		Figures between 18 and 24.9 indicate normal weight.
		Figures between 25 and 26.9 indicate overweight.
		Figures between 27 and 40 indicate
		varying degrees of obesity.

**Table 2:** Sample of data analyzed in the project.

Age	DxTx	FC	FR	Temp	Systolic	Diastolic	Smoke	BMI
20	118	75	21	37	122	86	1	34.6
21	174	84	15	36.7	94	76	0	24.7
22	124	86	18	36.7	112	85	0	30.7
22	160	88	18	36.9	111	78	1	23.4
19	112	58	17	36	112	75	0	22.7
23	125	63	13	37	115	82	0	28.8
21	127	78	15	35.6	135	87	0	25.3
18	103	77	18	36.4	130	107	1	19.4
24	147	79	17	36.2	116	78	0	19.7

**Table 3:** Data classification.

Ranking	Expected Value	Simulated Value
Class 1	0.25	0.2819
Class 2	0.50	0.3721
Class 3	0.75	0.7439
Class 4	1	0.8682

**Table 4:** Record of student information.

			Glucose	Heart Frequency	Respiratory Frequency		Blood Pressure				
No. Control	Sex	Age	DxTx	FC	FR	Temp °C	n0, s1 TA	Systolic <120 mmHg	Diastolic <80 mmHg	smoke	IMC
1	H	20	118	75	21	37	122/86	122	86	Si	34.6
2	M	21	174	84	15	36.7	94/76	94	76	No	24.7
3	H	22	124	86	18	36.7	112/85	112	85	No	30.7
4	M	22	160	88	18	36.9	111/78	111	78	Si	23.4
5	H	19	112	58	17	36	112/75	112	75	No	22.7
6	H	23	125	63	13	37	115/82	115	82	No	28.8
7	M	21	127	78	15	35.6	135/87	135	87	No	25.3
8	H	18	103	77	18	36.4	130/107	130	107	Si	19.4
9	M	24	147	79	17	36.2	116/78	116	78	No	19.7
10	H	21	130	66	19	36.3	129/66	129	66	Si	19.7
11	H	20	116	98	17	32.6	140/118	140	118	No	35.9
12	H	22	122	91	>12	36.7	127/92	127	92	No	27.7

**Table 5:** Blood pressure category, defined by Binu, D.; Rajakumar, B., in [25].

BLOOD PRESSURE CATEGORY	SYSTOLIC mm Hg (Upper Number)		DIASTOLIC mm Hg (Lower Number)
NORMAL	LESS THAN 120	AND	LESS THAN 80
ELEVATED	120–129	AND	LESS THAN 80
HIGH BLOOD PRESSURE (HYPERTENSION STAGE 1)	120–139	OR	80–89
HIGH BLOOD PRESSURE (HYPERTENSION STAGE 2)	140 OR HIGHER	OR	90 OR HIGHER
HIGH BLOOD CRISIS (consult your doctor inmediately)	HIGHER THAN 180	AND/OR	HIGHER THAN 120

**Table 6:** Analyzed information.

Sbp	Gender	Married	Smoke	Exercise	Age	Weight	Height	Overwt	Race	Alcohol	Trt	Bmi	Stress	Salt	Chldbear	Income	Educatn
133	F	N	N	3	60	159	56	3	1	2	0	35	2	2	2	2	2
115	M	N	Y	1	55	107	65	1	1	2	0	17	2	2	1	3	2
140	M	N	Y	1	18	130	59	2	1	1	0	26	3	2	1	1	3
132	M	Y	N	2	19	230	57	3	2	3	1	49	3	3	1	1	2
133	M	N	N	2	58	201	74	2	1	3	0	25	2	2	1	2	3
138	F	N	N	3	55	166	167	2	1	1	1	25	2	1	3	2	3
133	F	Y	N	1	22	188	66	3	1	3	1	30	3	1	3	1	1
67	F	Y	N	3	52	123	67	1	1	2	0	19	2	3	2	3	2
138	M	Y	N	1	46	106	73	1	1	3	1	13	2	2	1	2	1

**Table 7:** Blood pressure values.

Category	Blood Pressure	Systolic Pressure
Clase_1	Normal	Minor 120
Clase_2	High	120–129
Clase_3	Stage 1 Hypertension	130–139
Clase_4	Stage 2 Hypertension	140 or higher

**Table 8:** Correlation of variables Class1_Systolic pressure normal.

Normal							
	Age	DxTx	FC	FR	Temp °C	IMC	Systolic
Age	1						
DxTx	0.042692	1					
FC	0.098773	0.230647	1				
FR	0.075499	−0.03204	0.083564	1			
Temp °C	−0.01297	−0.03423	0.085965	0.077646	1		
IMC	−0.02441	0.010178	−0.00661	0.13376	−0.02438	1	
Systolic	−0.08638	0.11035	−0.14349	0.094649	−0.01691	0.131297	1

**Table 9:** Values obtained from the ANN with trainlm/newff.

net=newff(minmax(inputs),[14 50 20 4],{’tansig’,’logsig’,’logsig’,’purelin’},’trainlm’)			
	Tests	Correct	Clasification
Clase_1	5	3	60%
	10	8	80%
	15	13	87%
Clase_2	5	4	80%
	10	8	80%
	15	14	93%
Clase_3	5	3	60%
	10	7	70%
	15	6	40%
Clase_4	5	5	100%
	10	10	100%
	15	15	100%

**Table 10:** Values obtained from ANN with feedforwardnet/trainlm.

net = feedforwardnet([10 25 15 4]){’tansig’,’ tansig ’,’ tansig ’,’ tansig ’},’trainlm’)			
	Tests	Correct	Clasificaction
Clase_1	5	5	100%
	10	10	100%
	15	15	100%
Clase_2	5	4	80%
	10	6	60%
	15	12	80%
Clase_3	5	4	80%
	10	7	70%
	15	12	80%
Clase_4	5	5	100%
	10	10	100%
	15	15	100%

**Table 11:** Comparative table of references [30,31,32,33].

Machine Learning Method	Comments
Machine Learning SVM	This article mentions the use of SVM in combination with simple k; implies to obtain a lower order error and determine the tumour region by consolidating the inherent image structure progression [30] but does NOT mention its effectiveness and accuracy.
	The Support Vector Machine (SVM) algorithm can be used for classification and regression problems. However, SVMs are quite popular for relatively complex types of small to medium-sized classification datasets. In this algorithm, the data points are separated by a hyperplane and the kernel determines the appearance of the hyperplane. If we plot multiple variables on a normal scatter plot, in many cases, that plot cannot separate two or more classes of data. The kernel of an SVM is an important element, which can convert low dimensional data into a higher dimensional space, ref. [31]. The authors explain in a very limited way without indicating in what way it can be implemented [31].
	Cervical cancer can be diagnosed with the help of algorithms such as decision tree, logistic regression and support vector machine (SVM) [31].
	Several machine learning classification algorithms have been used in Predictive Model Selection (PMS), namely, support vector machine (SVM), decision tree classifier (DTC), random forest (RF), logistic regression (LR), gradient boosting (GB), XGBoost, adaptive boosting (AB) and k-nearest neighbour (KNN). The authors refer to them theoretically but do not support their efficiency in their implementation [31].
	The paper presented a skin cancer detection system using a support vector machine (SVM), which helps in early detection of skin cancer disease. They used traditional image processing and feature engineering methods for effective feature selection and support vector machine (SVM) algorithms for feature classification [32].
	Performance evaluation was carried out using four different classifiers such as decision tree (DT), k-nearest neighbour (KNN), (KNN) tree, boosted decision tree (BT) and SVM. The classification was performed using the relevant vector machine and SVM classifier, which achieved 92.4% [32]. The authors make a comparison with other classifiers.
Naive Bayes machine learning	This type of machine learning is not mentioned in any of the cited and suggested articles.
Machine Learning ANN	The authors conducted a survey-based study on cervical cancer detection, including a performance analysis to determine the accuracy of several distinctive types of architecture in an artificial neural network (ANN), where the ANN was used to identify cancerous, normal and abnormal cells. Ref. [31] The authors theoretically present the use of ANNs to identify cancerous, normal and abnormal cells.

**Table 12:** Comparative table of references [34,35,36,37].

Machine Learning Method	Comments
Machine Learning SVM	SVM models are characterized by processing both linear and non-linear data. The model aims to draw decision boundaries between data points of different classes and separate them with the maximum margin [34].
	SVM slightly outperforms ANN in recognition using one dataset. The exact reason for this improvement is difficult to pinpoint and could simply be due to better parameter selection or the diverse and non-linear nature of the dataset, or both. It could also be due to the fact that SVM converges to a global minimum and allows for better noise tolerance.
	The vector machine is a very popular supervised machine learning technique (with a predefined target variable) that can be used as a classifier and as a predictor [34].
	https://www.ijcaonline.org/archives/volume183/number43/jijji-2021-ijca-921837.pdf (accessed on 29 May 2022). [35]
	SVM has the highest accuracy 0.9985. This part shows three machine learning algorithms, which are KNN, Bayesian and SVM, applied for the same objective [35].
	https://mdpi-res.com/d_attachment/diagnostics/diagnostics-09-00178/article_deploy/diagnostics-09-00178.pdf?version=1573124691 (accessed on 29 May 2022). [36]
	- SVM can achieve better generalization ability in small sample classification tasks, and has been widely used in medicine.
	- Theoretically, SVM can achieve optimal classification.
	- SVM can be well applied to pattern recognition, time series prediction and regression estimation, among others [36].
Naive Bayes machine learning	https://iopscience.iop.org/article/10.1088/1757-899X/1022/1/012072/pdf (accessed on 29 May 2022). [37]
	Naive Bayes is a simple but effective classification technique based on Bayes’ Theorem. It assumes independence between predictors, i.e., the attributes or features must be uncorrelated or unrelated to each other. Even if there is dependence, all these characteristics or attributes contribute independently to the likelihood.
	- In ensemble modeling, two or more related but different analytical models are used and their results are combined into a single score. An ensemble of SVM, KNN and ANN have been used to achieve an accuracy of 94.12%. The majority vote-based model as demonstrated by Saba Bashir et al. [26], which is composed of Naïve Bayes, Decision Tree and Support Vector classifiers, gave an accuracy of 82%, a sensitivity of 74% and a specificity of 93% for and a specificity of 93% for the ICU heart disease dataset. an ensemble model, consisting of Gini index, SVM and Naïve Bayes classifiers, was used which provided 98% accuracy in predicting syncope disease [37].
Machine Learning ANN	https://www.nature.com/articles/s41598-018-24926-7.pdf?origin=ppub (accessed on 29 May 2022).
	A comparison is made between the SVM and ANN method implemented in pattern recognition, specifically in the detection of insects contaminating food [34].

## Data Availability

Not applicable.

## Abbreviations

The following abbreviations are used in this manuscript:

AHT	Arterial Hypertension
AI	Artificial Intelligence
CIDIT	Centro de Investigación, Desarrollo e Innovación Tecnológica
ECITEC	School of Science and Technology
mg/gL	milligrams per decilitre of blood
HR	Heart Rate
IMC	Body Mass Index
INSP	National Institute of Public Health
MLP	The Multilayer Perceptron
PAHO	PanAmerican Health Organization
RF	Respiratory Frequency
UABC	Autonomous University of Baja California
WHO	World Health Organization
